# Comparative analysis of the biomechanical behavior of two different design metaphyseal-fitting short stems using digital image correlation

**DOI:** 10.1186/s12938-020-00806-y

**Published:** 2020-08-19

**Authors:** I. Tatani, P. Megas, A. Panagopoulos, I. Diamantakos, Ph. Nanopoulos, Sp. Pantelakis

**Affiliations:** 1 Orthopaedic Department, University Hospital of Patras, Papanikolaou 1, Rio-Patra, 26504 Patras, Greece; 2 Laboratory of Technology and Strength of Materials, Department of Mechanical Engineering and Aeronautics, University of Patras, Patras, Greece; 3 Department of Computer Engineering & Informatics, University of Patras, Patras, Greece

**Keywords:** Total hip arthroplasty, Short stem, Digital image correlation, Finite element analysis, Experimental validation

## Abstract

**Background:**

The progressive evolution in hip replacement research is directed to follow the principles of bone and soft tissue sparing surgery. Regarding hip implants, a renewed interest has been raised towards short uncemented femoral implants. A heterogeneous group of short stems have been designed with the aim to approximate initial, post-implantation bone strain to the preoperative levels in order to minimize the effects of stress shielding. This study aims to investigate the biomechanical properties of two distinctly designed femoral implants, the TRI-LOCK Bone Preservation Stem, a shortened conventional stem and the Minima S Femoral Stem, an even shorter and anatomically shaped stem, based on experiments and numerical simulations. Furthermore, finite element models of implant–bone constructs should be evaluated for their validity against mechanical tests wherever it is possible. In this work, the validation was performed via a direct comparison of the FE calculated strain fields with their experimental equivalents obtained using the digital image correlation technique.

**Results:**

Design differences between Trilock BPS and Minima S femoral stems conditioned different strain pattern distributions. A distally shifting load distribution pattern as a result of implant insertion and also an obvious decrease of strain in the medial proximal aspect of the femur was noted for both stems. Strain changes induced after the implantation of the Trilock BPS stem at the lateral surface were greater compared to the non-implanted femur response, as opposed to those exhibited by the Minima S stem. Linear correlation analyses revealed a reasonable agreement between the numerical and experimental data in the majority of cases.

**Conclusion:**

The study findings support the use of DIC technique as a preclinical evaluation tool of the biomechanical behavior induced by different implants and also identify its potential for experimental FE model validation. Furthermore, a proximal stress-shielding effect was noted after the implantation of both short-stem designs. Design-specific variations in short stems were sufficient to produce dissimilar biomechanical behaviors, although their clinical implication must be investigated through comparative clinical studies.

## Background

Total hip arthroplasty (THA) is arguably considered a reliable procedure to provide pain relief, restore function and improve quality of life in patients with advanced hip osteoarthritis [1–9]. While in the past, the procedure was mainly reserved for fragile elderly patients, the success of the procedure has expanded its indications to relatively younger and more active patients [10], whose sole debilitating limitation in daily living is the affected hip joint. In order to cope with this new surge of popularity of THA, several efforts have been made to accelerate the rehabilitation process, maximize the longevity of the implants and eliminate morbidity related to a future revision procedure. Recent innovations are therefore mainly aimed towards reducing soft tissue damage and preserving native bone without compromising implant stability [11].

The reduction of strain in the bone caused by an adjacent load-carrying implant can lead to a subsequent reduction in surrounding bone density, causing the well-known stress-shielding phenomenon [12, 13]. There are conflicting data in the literature concerning the clinical significance of stress-shielding effect, with some authors advocating that the adverse implant-induced bone adaptation can compromise the longevity of cementless THA [14–16]. According to other studies, periprosthetic bone resorption commonly observed around cementless implants has not yet correlated with any increased risk of aseptic loosening or periprosthetic fracture [17–20]. Though it is not proven that stress-shielding effect is directly related to the survival of implants, an excessive bone loss around a primary prosthesis can complicate a potential revision procedure. Consequently, the preservation of proximal periprosthetic bone is considered a vitally important principle in THA, and different stem designs have been launched in an effort to preserve a physiological load transfer to the femur, thus eliminating stress-shielding effect.

A heterogeneous group of conservative femoral prostheses, called short stems, have emerged as an alternative to conventional stems, with differences in terms of design, biomechanics and method of fixation [21, 22]. Ideally, a short-stem implant should provide rigid primary fixation, extensive metaphyseal bone–implant contact for sufficient osseointegration and a more physiological load transfer reproducing a biomechanical behavior more similar to the physiological bone. However, the benefits of short femoral stems remain hypothetical and there is not a clear understanding of the influence of short-stem designs on bone biomechanics [23, 24].

Numerous biomechanical studies have been performed comparing different femoral stem design features in an attempt to find a way to eliminate the stress-shielding effect of the implanted femurs. Most studies indicate that the stiffer the implant, the more pronounced the stress shielding [25–28]. The following solutions have been proposed in an effort to reduce stems’ stiffness, including an optimum selection of constitutive materials [29–31], internal structure, geometry and shape [32–38].

**Fig. 1:**
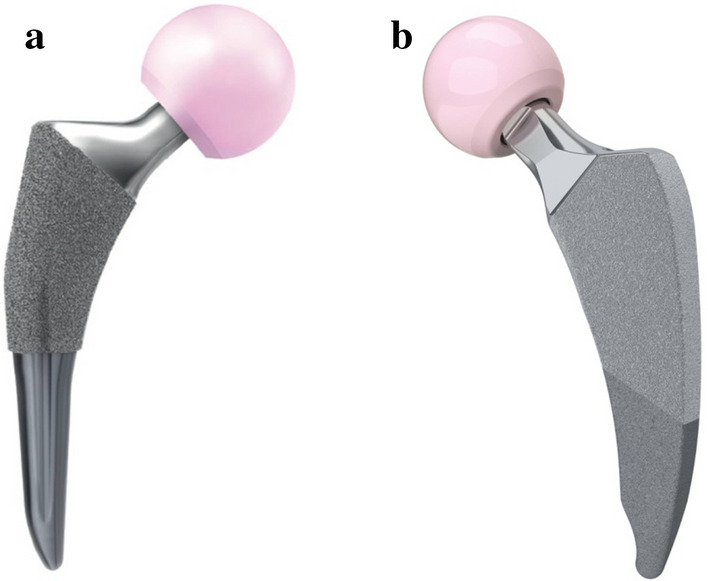
Femoral stems. **a** Trilock BPS stem. **b** Minima S stem

This study combines experiments and numerical simulations to investigate the biomechanical properties of two different design short stems, the TRI-LOCK Bone Preservation Stem (DePuy Orthopaedics Inc. Warsaw, USA) (Fig. 1a) and the Minima S Femoral Stem (Lima corporate Villanova di San Daniele, Italy) (Fig. 1b). According to the detailed classification system of short stems proposed by Gómez-García et al. [39] in 2016, the Tri-Lock Bone Preservation Stem (Tri-Lock BPS) and Minima S stems are classified as type C, meaning that they occupy the cervico-metaphyseal-diaphyseal area. In our point of view, an important issue that should be addressed is whether all variations of currently available metaphyseal-fitting short stems could offer similar load transfer characteristics before making any generalized recommendation for their overall use. The study’s hypothesis was that even these subtle variations, regarding stem length and geometric design between these two stems, which are classified within the same subgroup, may create different strain distribution patterns and thus dissimilar biomechanical behaviors. For this purpose, the transmission of forces as measured by cortical surface strain distribution in the proximal femur was evaluated using Digital Image Correlation (DIC), first on the non-implanted femur and then on the implanted femurs with the TRI-LOCK BPS and Minima S femoral stems in respect. The strain patterns of the non-implanted femur served as the control group. The DIC full-field strain patterns in intact and implanted composite femurs were compared and also the corresponding numerical models were developed and evaluated for their validity against mechanical tests.

## Results

### Comparison of DIC-measured strains between intact and implanted femurs

**Fig. 2:**
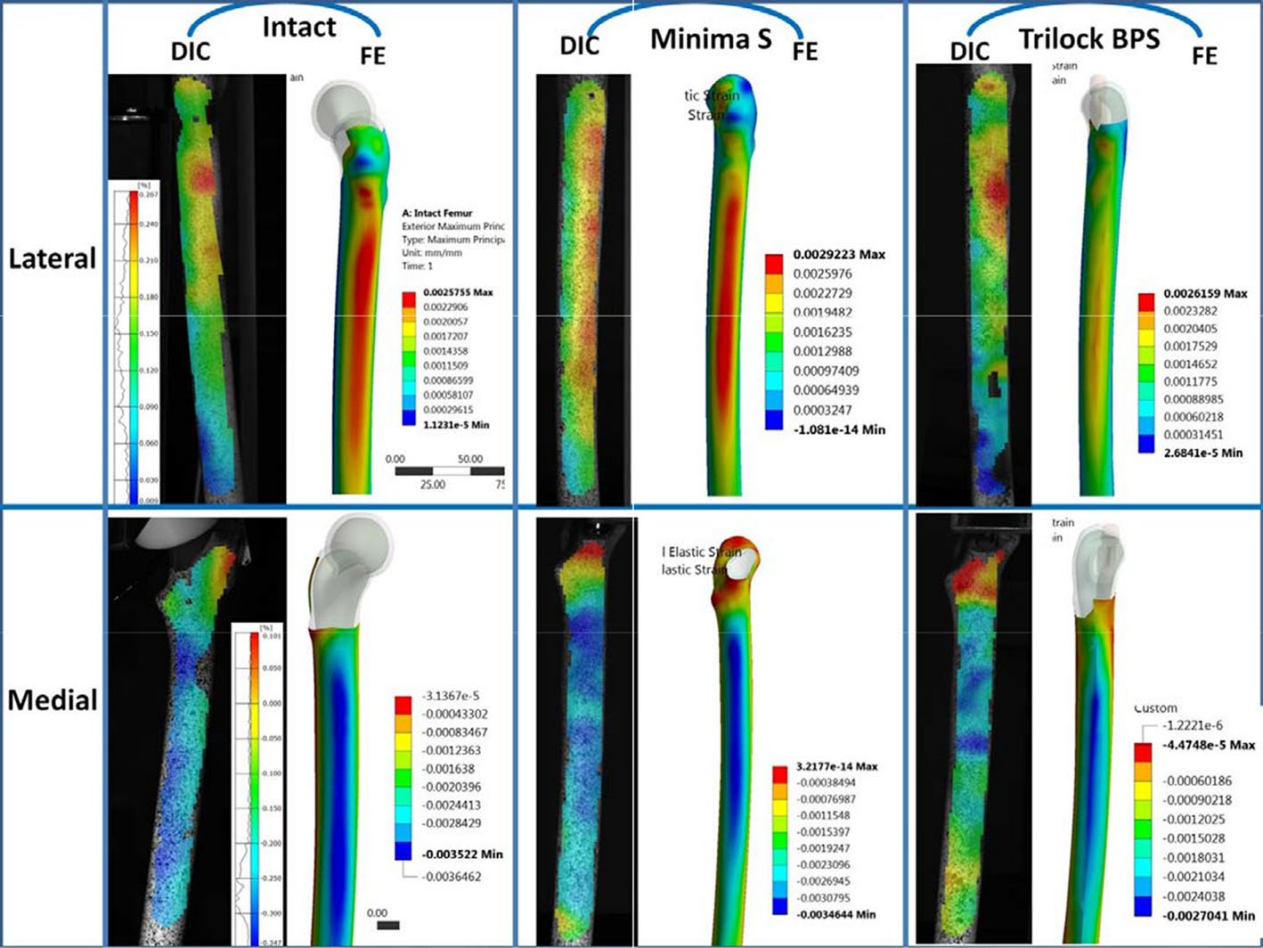
DIC-measured and FE-predicted strains for each specimen in each of the two fields of view

Before comparing DIC-measured strains between intact and implanted femurs, DIC strains of the several tests concerning the same case and loading level (1000 N was considered) were compared via linear correlation analyses. Slopes were close to 1 and thus the fields obtained from different repetitions showed a good agreement. Figure 2 depicts the typical numerical and experimental fields for the intact and implanted bones in each of the two fields of view. In the DIC analysis, the non-implanted femoral bone, serving as a reference model of healthy bone exhibited higher strain response to loading in the proximal medial area than any of the implanted femurs. The quantitative full-field strain analysis demonstrates a clear trend of increased strain along the longitudinal axis of the femur from proximal to distal at the medial side. In the proximal medial aspects of the femur (zones of interest M1, M2) there was a statistically significant decrease in the mean principal compressive strain of implanted bones compared to that of the intact femur. The percentage variance in the mean compressive strain at these proximal medial zones was greater for the Trilock BPS compared to the Minima S. At zones M3 to M7, implanted specimens exhibited a strain response most closely matching that of the intact femur. At these distal zones, the percentage variance of strain was less pronounced in both groups of implanted femurs.

**Fig. 3:**
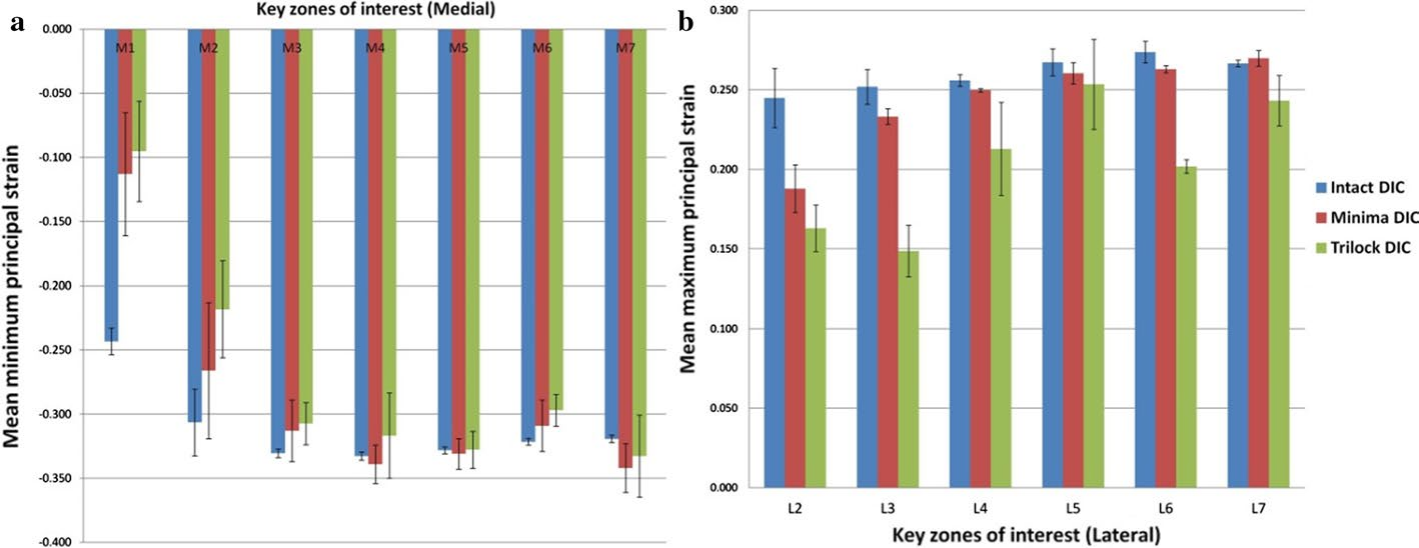
DIC-measured strain response to single-leg stance loading in **a** medial and **b** lateral measurement regions

At the L1 zone of interest, the attachment of the metallic blade to the greater trochanter prevented DIC equipment to visualize this zone, and hence the measured strain data are lacking from this part of the bone. On the lateral aspect of the femur a decrease in principal tensile strains was observed in the implanted femurs at almost all measurement zones compared to the intact bone. The decrease in strain response was more pronounced for the Trilock BPS implanted specimens and significant differences were observed compared to the Minima S at almost each measurement zone. Strain response at each zone of interest along the medial and lateral femoral cortex is presented in Fig. 3, with statistical analysis presented in Table 1.

**Table 1:** Percentage strain for each prosthesis compared to the control femur and strain comparison for each zone of interest, *p*-values

Key zone of interest	Implanted Minima S femoral stem		Implanted Trilock BPS femoral stem		Minima S to Trilock BPS (*p*-value)
	Percentage strain against controls (%)	Mann–Whitney test against controls (*p*-value)	Percentage strain against controls (%)	Mann–Whitney test against controls (*p*-value)	
Medial side					
M1	68.7	0.001	57.1	0.000	0.029
M2	84.1	0.005	69.2	< 0.0001	< 0.0001
M3	101.0	0.029	89.4	< 0.0001	< 0.0001
M4	102.5	0.529^a^	97.8	0.139^a^	< 0.0001
M5	99.1	0.459^a^	98.2	0.752^a^	0.153^a^
M6	98.3	0.13^a^	96.7	< 0.0001	0.000
M7	106.1	0.001	101.6	0.556^a^	0.003
Lateral side					
L2	87.5	< 0.0001	79.2	< 0.0001	0.935^a^
L3	89.1	< 0.0001	57.8	< 0.0001	< 0.0001
L4	98.2	0.000	82.2	< 0.0001	< 0.0001
L5	98.1	0.043	92.6	0.004	0.002
L6	95.4	0.000	81.2	< 0.0001	< 0.0001
L7	100.3	0.131^a^	87.7	< 0.0001	0.006

^a^Not significant

### FE model validation

**Fig. 4:**
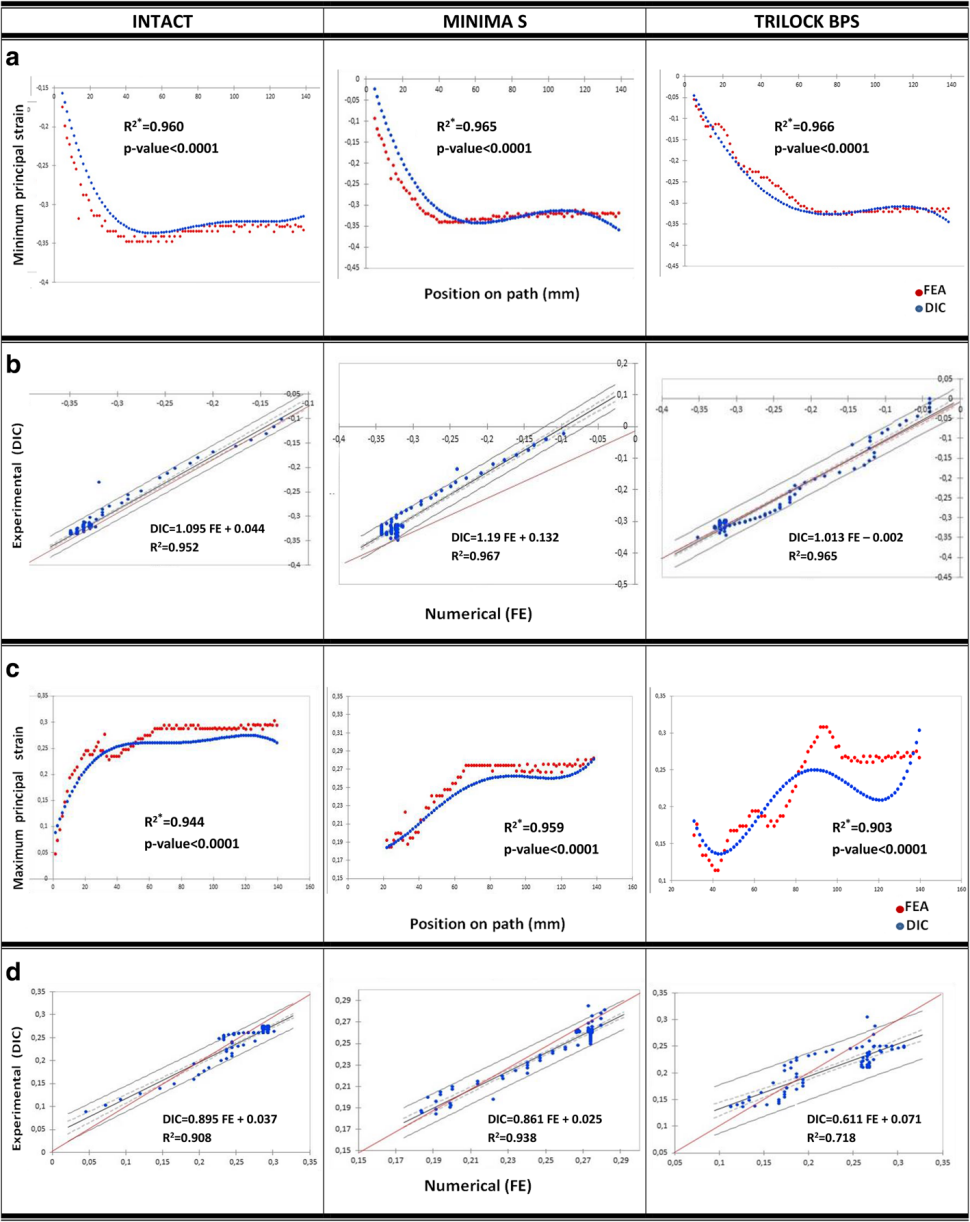
*Medial field of view.* ***a*** Best-fitting polynomial curves of the FE-predicted and DIC-measured strain data relative to the long axis of the bone for the intact and implanted bones with Minima S and Trilock BPS. **b** Linear regression analysis. *Lateral field of view.* **c** Best-fitting polynomial curves of the FE-predicted and DIC-measured strain data relative to the long axis of the bone for the intact and implanted bones with Minima S and Trilock BPS. **d** Linear regression analysis; *R*^2*^: coefficient of determination in polynomial regression, *R*^2^: coefficient of determination in linear regression analyses

The obtained best-fitting polynomial curves of the experimental and calculated strain data relative to the long axis of the bone are presented in Fig. 4a, c for the medial and the lateral side, respectively. The validation of the numerical models was conducted via linear correlation analyses of the measured and calculated principal strains for each construct (Intact Femur, Minima S and Trilock BPS implanted bones) in each field of view. More specifically, FE simulation results and experimental measurements concerning minimal strains at the medial path of the bone were compared for the intact, Minima S and Trilock BPS implanted bones, respectively (Fig. 4b). Similarly, analytically and experimentally derived maximum strains at the lateral path of the bone were compared for the same three cases as above (Fig. 4d). The numerical model validation relies on the reasonable agreement observed between the numerical and experimental data in the majority of cases with the exception of the Trilock BPS implanted femur in the lateral surface (slope = 0.611, *R*^2^ = 0.718).

## Discussion

This study was designed to investigate the biomechanical properties of two different design short stems, based on biomechanical testing and finite element models. These two methods should be combined to yield more precise measurements, as recommended by previous studies focusing on the evaluation of strain patterns induced after the implantation of a hip prosthesis [40, 41].

The DIC technique has been introduced into the field of biomechanics for accurate determination of surface strain in inhomogeneous, anisotropic, non-linear materials such as bone. The strain distribution patterns induced by different materials have been studied previously in the laboratory setting using strain gauge analysis [28, 42–45], photoelastic coating techniques [46, 47] and finite element analysis [48–50]. Traditionally, strain gauges have been considered the ‘gold standard’ in the experimental testing for evaluation of the biomechanical behavior of bone in vitro since their introduction in the late 1950s [51]. Although they are known to be reliable and despite their widespread use, strain gauges require surface preparation and provide strain results only at the restricted area of application. This lack of full-field strain data is considered to be a limitation of the technique. Digital image correlation is an optical full-field technique that allows noncontact, three-dimensional deformation measurements of objects subjected to external loads. To date, DIC has been used in many applications in biomechanics, such as a measurement tool of strains on bone surface with or without validation of finite element corresponding models [24, 31, 52–59] and also to evaluate relative micromotion between the implant and the surrounding bone [24, 60]. In this study, we employed the three-dimensional DIC measurement technique to find the differences in strain patterns generated in composite femurs implanted with two different design femoral prostheses. The results showed that the DIC technique captured the strain on the bone surface well, providing full-field plots for each case, as opposed to application point results which would have been achieved using strain gauges.

The clear distal load transfer after the implantation of both short stems demonstrated in this study is congruent with previous studies, which have found a similar distally shifting load distribution pattern as a result of implant insertion [24, 61, 62]. Despite the fact that short-stem femoral implants have displayed a better biomechanical behavior preserving a strain distribution closer to the intact bone according to previous studies [24, 28, 42, 43, 63–65], the implantation of a stiffer material absorbs the load and transfers it distally, leaving the proximal region of the calcar somewhat stress-shielded. The data presented here demonstrate that strain shielding and proximal unloading of the femur occurred even when using short-stem implants. Although there is a lack of evidence that stress-shielding effect could directly influence the clinical results, it is of predominate concern that a resorption of proximal femoral bone stock may negatively affect the stability and long-term survival of femoral implants [28, 66, 67].

The results of the current study have potential clinical implications. While strain distribution patterns after the implantation of femoral implants in total hip arthroplasty have been previously investigated, this study determined quantitatively the specific strain data derived by two short femoral stems, which belong to the same category of cervico-metaphyseal-diaphyseal short stems. It seems that, even these subtle variations regarding stem length and geometric design are sufficient to produce significant strain changes. Although the clinical implication of the aforementioned differences in biomechanical behavior cannot be predicted, the clinicians must be really hesitant before making general recommendations about the clinical results of short femoral implants, even if they belong to the same category. Taking into consideration that the reduced strain in a region of bone is the definition of stress shielding, the patterns of strain distribution derived after the implantation of these stems could be correlated with the clinical results of future in vivo studies focusing on the bone remodeling response surrounding these implants.

Quantitatively, full-field strain distributions were in good agreement to the FE-predicted strain patterns in the majority of cases. Although a reduced correlation was observed for the Trilock BPS implanted femur in the lateral surface, the discrepancies could be explained by the fact that high spread values were obtained in this case.

This study, however, has certain limitations. At first, composite femoral bones were chosen instead of cadaveric human specimens. We acknowledge that the strain patterns induced after mechanical testing of these specimens are not equivalent to in vitro cadaveric models or in vivo clinical conditions. However, mechanical testing on synthetic femurs is considered a valid method of studying the biomechanical behavior of implanted femurs [68, 69]. According to previous studies, composite femurs have been independently tested and shown a biomechanical behavior similar to that of human cadaveric specimens during loading [68, 70, 71]. The composite bones present a lot of advantages compared to cadaveric specimens, including minimal inter-specimen variability, consistent material properties, high availability and low cost. Additionally, composite bones are independent from parameters that can substantially alter their properties, such as storage method, air temperature and humidity and time from harvest [70]. In this study, where a direct comparison between two implant systems was made, the proven low inter-specimen variability of composite femurs can provide more consistent results, eliminating confounding factors.

In the presented experimental testing setup and in accordance with previous reports [26, 31, 64, 72] the physiological load applied to the femoral bone was reduced to a simpler configuration, where only the abductors were included. Despite the complexity of the biomechanics involving the hip, recent in vitro biomechanical studies evaluating femoral prostheses tend to be as simple as possible, not fully accounting for the numerous soft-tissue interactions which actively contribute to hip joint stability and femoral loading characteristics. Furthermore, abductor muscle forces have been demonstrated to exert the greatest impact on strain patterns in the proximal femur and thus the other soft tissues could be ignored in a first approximation [73].

In this study, the phase of gait to be simulated was chosen to be the single-leg stance that represents a simplified model of the natural physiological loading state. The fact is that there is no general agreement about the optimal loading condition for the evaluation of changes in strain patterns on the proximal femur [74]. This simplified loading phase already described in the literature [75] combines simultaneous compression and bending forces and results in a more physiological loading condition than isolated compression or bending loading.

Experimental studies evaluating strain patterns in femoral bones after insertion of femoral prostheses are valuable in the assessment of their biomechanical behavior. Nevertheless, we acknowledge that neither experimental studies nor numerical analyses can uncritically predict the clinical performance of an implanted material. We are also aware that cortical strain measurement cannot directly reflect the in vivo performance because composite models represent a nonviable bone without the capacity to be remodeled. For this purpose, a simultaneous clinical study is already running, aiming to assess the remodeling process around these specific implants in vivo, by means of a detailed radiological analysis [76]. Nevertheless, this testing setup provides useful data for the biomechanical behavior of these implants, which in turn may indicate their clinical performance in the first postoperative period.

Concerning finite element models, the usual limitations are present. The material properties of bone are orthotropic rather than isotropic, as specified in this finite element model. Nevertheless, the developed numerical model could be considered valid and reliable as provided by the reasonable agreement observed between the numerical and experimental data.

## Conclusions

This study provides evidence to support the use of DIC technique as a preclinical evaluation tool of the biomechanical behavior induced by different implants. The results of this study also support DIC’s potential for biomechanical FE model validation. Design differences between Trilock BPS and Minima S conditioned different strain pattern distributions and thus our primary hypothesis that design-specific variations in these specific short stems are sufficient to produce dissimilar strain patterns was confirmed. An obvious decrease of strain in the medial proximal aspect of the femur was noted for both stems, demonstrating that proximal unloading of the femur could not be avoided. At the lateral surface, both implanted specimens exhibited a persistent decrease in principal tensile strains at almost all measurement zones compared to the intact bone. Nevertheless, alteration in strain patterns induced after the implantation of the Trilock BPS stem was greater compared to the Minima S stem at all regions of interest on the lateral cortex. Being aware that the findings of this study could not be used uncritically to predict the in vivo performance of these femoral implants, our ongoing randomized, clinical study aims to add information regarding the clinical effects of the differences observed in femoral strain patterns between the two prostheses.

## Methods

### Experimental study

The experimental work was undertaken in the Laboratory of Technology and Strength of Materials at the Department of Mechanical Engineering and Aeronautics. Short stems with predominantly metaphyseal fixation, such as the TRI-LOCK Bone Preservation Stem and the Minima S Femoral Stem were chosen for the purpose of the study.

*Tri*-*Lock BPS* is a short tapered-wedge stem. It is made of titanium alloy with a highly porous pure titanium (“GRIPTION^®^”) coating on the proximal 50% portion that is engineered to provide an enhanced coefficient of friction when compared to POROCOAT^®^ porous coating, which is on the original Tri-lock stem. Compared to its clinically successful predecessor, the Tri-Lock stem, the BPS stem is shorter, has a narrower distal segment, and features a curved distal tip. It is available in 13 stem sizes (size 0–12/length 95–119 mm) with standard and high offset options for all stem sizes. The high offset option provides direct lateralization, increasing offset without affecting either the leg length or the neck-shaft angle.

*Minima S Monolithic Femoral Stem* is a short, curved, four tapered proximally porous-coated titanium femoral stem with 12 stem sizes (size 1–12/length 82–118 mm) in standard and lateralized configuration available. The standard versions have a neck–shaft angle of 134°, while the lateralizing versions have a neck-shaft angle of 131°.

Although both stems belong to the same short stem family, Minima S stem is even shorter compared to Trilock BPS, has an anatomic shape following the natural curvature of the medial calcar, preventing breach of the greater trochanter and a medially sidecutted tip to reduce the risk of contact with the cortical medial wall.

A total of seven fourth-generation medium composite femurs from Sawbones Europe (Malmö, Sweden) with identical design and material properties were used, as previous described in similar biomechanical studies [66, 70, 77–81]. For each prosthesis, three different composite femoral bones were randomly allocated to receive either the Tri-Lock BPS or the Minima S Femoral Stem. The intact femur was used as a bench top experimental estimate of the non-implanted state.

To provide a proper primary fixation and restore the hip biomechanics as accurately as possible, the choice of the correct implant size and offset is imperative. Changes of the neck offset after the implantation of the femoral stem relative to the intact reference bone could have an influence to the moment arm of the force applied on the femoral head, altering the strain distribution on the proximal femur. To account for these critical parameters, implants’ templates provided by the manufacturers (DePuy Orthopaedics Inc. Warsaw, USA and Lima corporate Villanova di San Daniele, Italy) were superimposed on calibrated radiographs of the intact femurs. Reference points were the femoral axis and the center of the femoral head, marked by means of a best-fitting circle to the intact femoral head. The implant size was determined as a size 4 high offset with a − 2, 28 mm diameter femoral head for the Trilock BPS stem. The size of 4 high offset with a − 4, 28 mm diameter femoral head was chosen for the Minima S femoral stem.

For the experimental preparation, the distal condyles of the intact femur were embedded into a steel cylinder using an ultra-low viscosity casting resin (Smooth-Cast Urethane Series 300 potting material, Smooth- On Inc, Easton, PA, USA). For a standardized embedding procedure, a custom alignment fixture was manufactured based on a previous reported femur-aligned reference system [82]. The embedding procedure was performed ensuring that the central axis of the femur through fossa piriformis coincided with the central axis of the cylinder and the posterior condylar surface was used for rotational alignment. For the correct positioning of the femur at the correct directions a system of arms, a laser level and a goniometer were used. Using the customized fixture, the femurs were positioned neutral on the sagittal plane and angled at 11° of adduction in frontal plane, which corresponded to the physiological inclination during single-leg stance [83].

Six different composite femoral bones (three for each prosthesis) were prepared to accommodate the prostheses and all implantations were performed by the same investigator. Each prosthesis was implanted into the prepared femur with a tight fit, without macro-movement evident when a compressive force was applied. The accuracy of implantation of the femoral stems in the composite femurs in terms of correct implant size and positioning was verified using calibrated radiographs of both the intact and implanted femurs. The radiographs of the best-fitting template-matched radiographs of the intact femurs were superimposed on those of the implanted femurs (see Additional file 1). After the radiographic evaluation, the implanted femurs were fixed into the steel cylinder using the same embedding technique as described above. The proximal end of each femur was then prepared for DIC compatibility. For the creation of the speckle pattern, the surface of each specimen was initially covered using matte white color. To form a thin and uniform background, the femurs were sprayed from a distance of 50 cm and left to dry for 10 min before black speckles were applied. Then, the random speckle pattern was created using matte black commercial spray paint can. In the resulting speckle pattern, the speckle size was 0.7–1.0 mm.

**Fig. 5:**
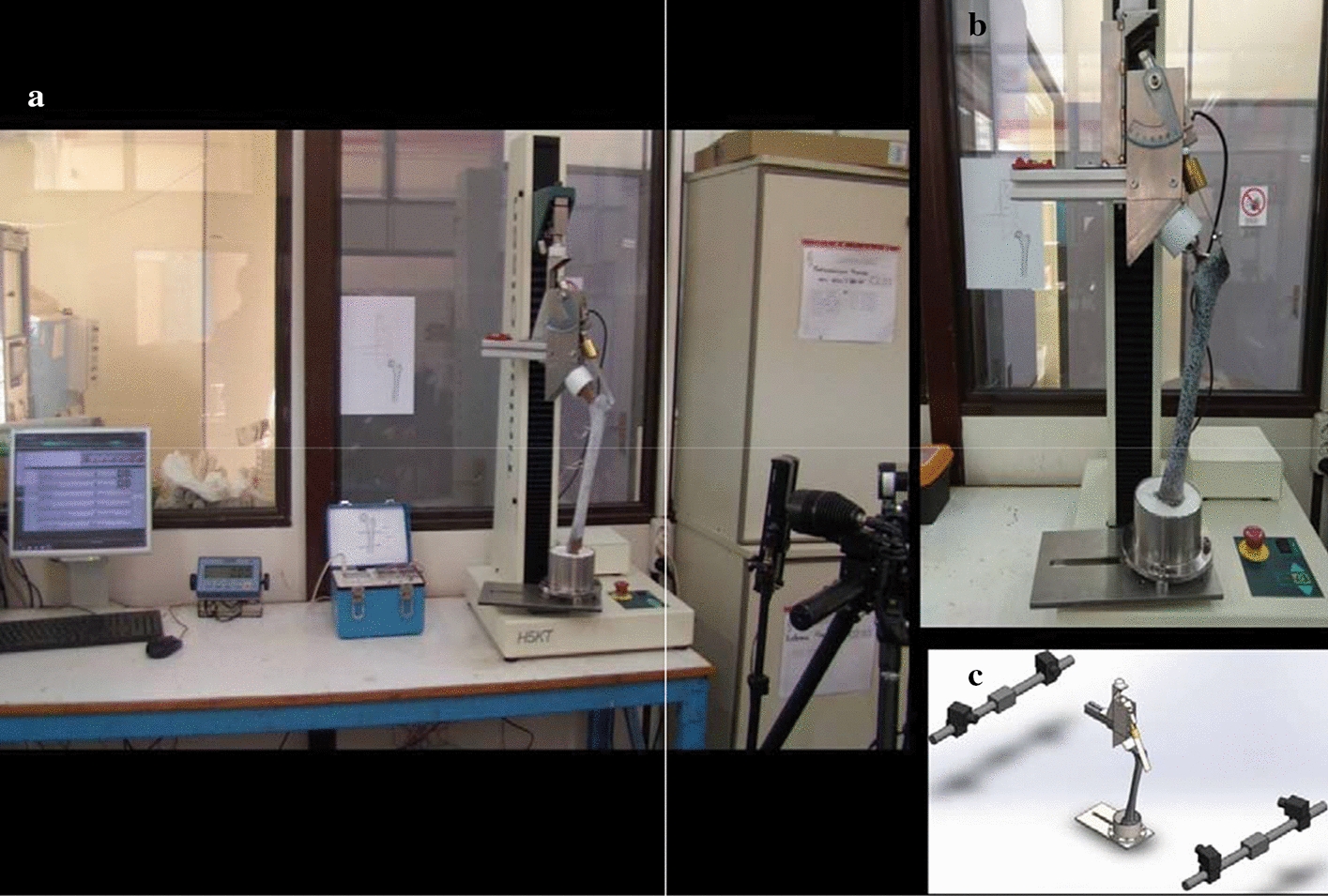
**a** Mechanical test setup; **b** detailed view of the experimental test setup showing the implanted femur within the customized loading apparatus allowing proximal loading via a compressive joint reaction force at the femoral head paired with a tensile force applied through the abductor plate on the greater trochanter; **c** configuration of the data capturing cameras

A custom-made mechanic jig was designed and manufactured according to the standardized protocol for testing conditions during functional validation of hip prostheses reported by Cristofolini and Viceconti [83] (Fig. 5). It consisted of a metal beam with an acetabular component properly attached to its undersurface, creating an articulation with the femoral head. The acetabular cup containing the femoral head had an inclination of 45° and 0° anteversion and its center was positioned 110 mm lateral to the load axis. The jig was supported on the femoral head through the acetabular cup and was attached medially to the load cell of the testing machine (Tinius Olsen electro-mechanical testing machine). The testing machine is computer-controlled and can apply tensile or compressive forces up to 5 kN. A system of cross rails were attached to the testing machine to guarantee that only a vertical force was applied to the cantilever device, avoiding undesired horizontal forces and moments. A system of rulers and goniometers allowed the position and direction of the forces to be controlled with an accuracy of ± 0.5 mm and ± 0.5°, respectively.

In the experimental fixture, a metallic blade was adhesively bonded at the lateral aspect of the greater trochanter, formed an angle of 40° to the femoral diaphysis, which simulated the pelvis. The test fixture was designed to provide a compressive force to the femoral head through the acetabular cup and a tensile force simulating the contraction of the abductors to the greater trochanter. A bolt was utilized to control the force and a load cell was used to monitor the force exerted by the abductors under loading condition. For the geometry of the specific loading fixture, which is based on the study of Cristofolini [83], the contraction force had to be 1.62 times the vertical force. Thus, at every load level applied and due to the deformation of the femur, the bolt has been used to adjust the force exerted by the abductors to the desired level (from 0 to 1620 N). The gait phase to be simulated was chosen to be the single-leg stance at the moment immediately after heal strike when the highest hip joint load acts [84, 85].

The non-implanted and implanted femurs were each placed into the custom-made jig, which was again mounted in the load cell of the testing machine. Loads were applied on the head of the intact composite femur and on the 28 mm metal head prostheses of the implanted bones through the acetabular cup. During the tests, the specimens were initially loaded with 100 N and the load was increased by increments of 100 N up to a total of 1000 N. Each craniocaudal load was applied three times and the strain patterns were recorded at the maximum load level of each loading cycle.

Experimental analysis was based on 3-dimensional optical measuring system, the Aramis 5 M DIC system (GOM mbH, Braunschweig, Germany). DIC measurements were performed using two different cameras positions capturing opposite femoral surfaces, the lateral (tension) and the medial (compression) surface of the bone. 3D DIC system consisted of a stand that provided stability of the cameras, an image recording and power control unit and the data processing software. The cameras’ setup used lenses with a focal length of 17 mm. The cameras were positioned at a relative angle of 25° and at a measuring distance of 875 mm, which led to a resolution of the captured images of 2448 × 2050 pixels. The field of view using the specific setup was 400 × 330 mm. The facet parameters of the DIC software were adjusted (facet size 19 × 19 pixels, facet step 15 × 15 pixels) and a post-processed filter was applied to achieve reliable results, reducing the noise affecting strain measurements. Linear strain calculation has been applied with three adjacent points used in the strain calculation.

The non-implanted and implanted femurs were each placed into the testing machine and load up to 1000 N was applied according to the configuration described above. Images of speckle patterns were captured for the intact and implanted bones at unloaded and under 1000 N of compression loading conditions. The images obtained were analyzed using the Aramis code. The intact composite femur and each of the six prepared femurs were tested in this way. Three trials were repeated for each specimen (intact and 3 composite bones implanted with each prosthesis) in each field of view. DIC strains between the experiments for each construct were compared among themselves to assure repeatability. After repeatability was confirmed, DIC-measured strains from the last experimental repetition of each construct (intact, implanted bone with Trilock BPS and implanted bone with Minima S femoral stem) were used for the comparison of DIC-measured strains between intact and implanted femurs. The measured variables were the minimum principal strain on the medial femoral side and the maximum principal strain on the lateral cortex.

### Numerical analysis

The geometrical model of the femoral Sawbone #3908 (Femur—Medium left/reference part #3403) was used in this study. It has been used as the base for the development of the finite element (FE) model. This geometrical model consists of three parts; the cortical bone and the proximal and distal cancellous cores. The average element size was set to 3 mm following a mesh-convergence study. The geometry of the two implants used in the present study has been acquired using the Hexagon Metrology Romer Absolute Arm equipped with an external high performance HP-L-20.8 laser scanner. The results of laser scanning were converted into 3-D solid models with the aid of a hybrid system using the CAD Software Catia V5 R 20. The geometry of the femur, after the neck osteotomy and before the implantation of each stem, has been created by the application of a Boolean operation, subtracting a cylindrical volume at the area of the femoral head. After modeling the femur, virtual implantation was performed positioning the implant in an orientation that replicated the tested specimen. Implant positioning in the model was based on the orthogonal photographs provided by the cameras during experimental setup and also the post-implantation antero-posterior and lateral radiographs. Parametric detailed 3D FE models of the femur and the implants have been developed using the commercially available ANSYS FE code. Tetrahedral elements have been used to develop the FE mesh, as they have been shown to discretize the femur and implants complicated geometries more efficiently than cubic hexahedral elements. Different linear material properties have been used to simulate the cortical and cancellous material of the bone. The femur consists of two different materials: short fiber-filled epoxy for the simulated cortical bone and solid rigid polyurethane foam for the simulated cancellous bone. Based on the data provided by the manufacturer, linear elastic, isotropic and homogeneous material properties were applied to the composite bone, with the simulated cancellous and cortical bone having Young’s modulus *E* = 155 MPa and *E* = 16.7 GPa, respectively. The Young’s modulus of both femoral stems constructed by titanium alloy (Ti6Al4V) was set as 110 GPa and the Poisson’s ratio for all materials was set as 0.3. Fully bonded contact conditions were used between all the components of the tested construct.

For verification purposes, the FE model of the non-implanted femur was validated against the mechanical tests on intact bones. The modeled implanted femurs were used thereafter to calculate strains at the medial and lateral femoral surface in order to identify highly stressed areas. These strain predictions were compared with those developed during mechanical testing of the two implanted stems for validation.

### Statistical analysis

**Fig. 6:**
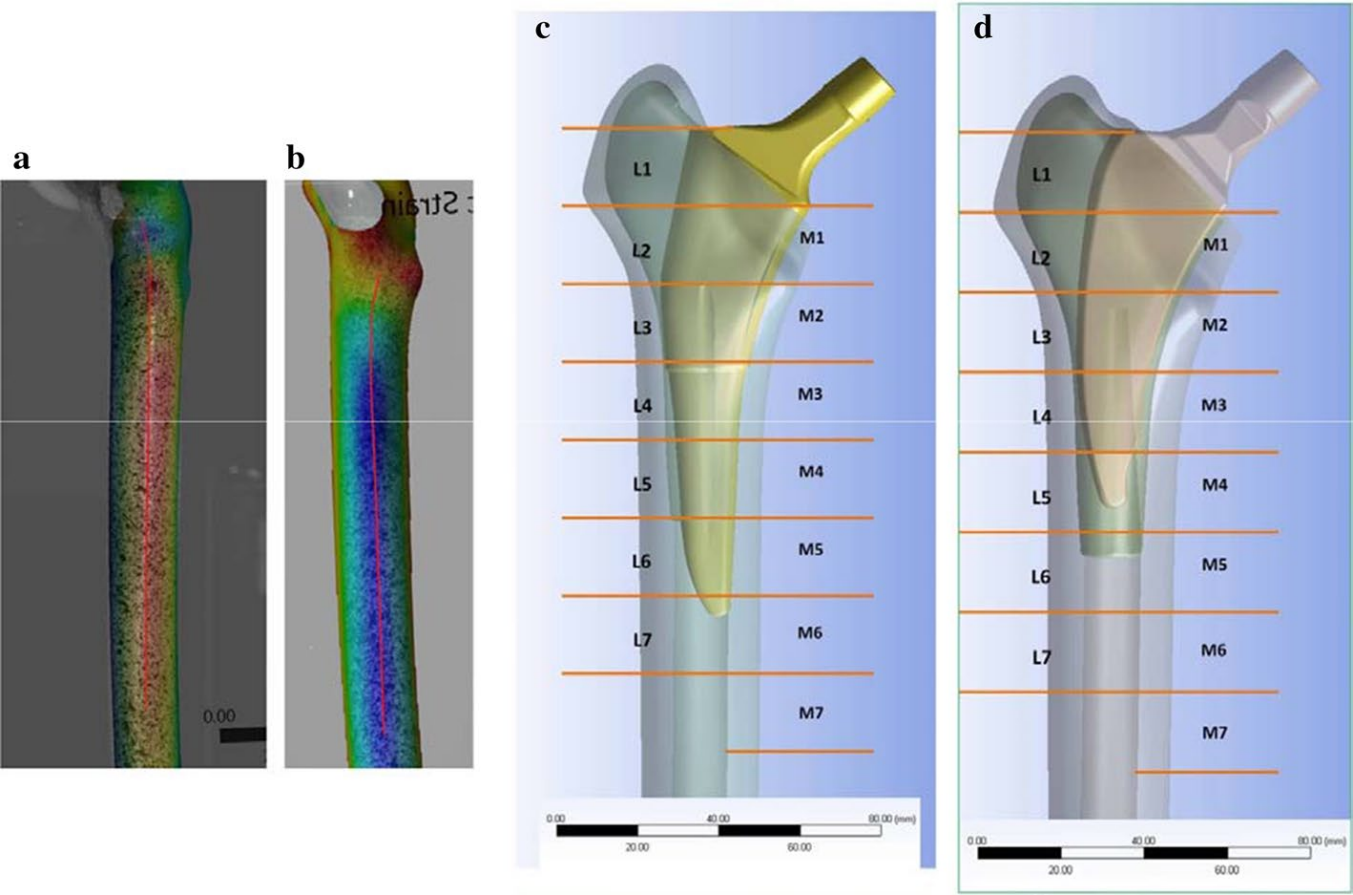
Comparison lines depicted on the DIC prepared surfaces, superimposed by their corresponding FE fields: **a** lateral surface; **b** medial surface. Key zones of interest at 2-cm increments along the long axis of the femur within each of the two fields of view; **c** Trilock BPS stem; **d** Minima S stem

For statistical analysis, two lines have been defined on the same part of each femur; one at the medial surface of the bone and one at the lateral surface. The position and direction of the comparison lines was based on well defined bony landmarks, the anatomical femoral axis and geometrical length measurements. These data were used to find comparable point coordinates in the DIC vs DIC or DIC vs FE corresponding fields (Fig. 6a, b). Key equal zones of interest within each of the two views (medial and lateral) were selected at 2-cm increments along these lines for quantitative strain comparison between experimental groups. Each line was divided in seven equal sections, designated as M1–M7 in medial side and L1–L7 in lateral side (Fig. 6c, d). Statistical analysis was performed utilizing Statistical Package for Social Sciences (SPSS) software (IBM SPSS Statistics version 25). Descriptive analysis was carried out for the intact and implanted femurs, at first globally along the medial and lateral femoral line and then for each key zone of interest, providing standard statistical parameters and the regression curves of strains. Modeling strain error as having a normal distribution was tested using Shapiro–Wilk test. The hypothesis of normality was rejected in 35 of the 72 cases at significance level of 5%. For this reason, a non-parametric Mann–Whitney *U* test with confidence level 95% (*a* = 0.05) was used to determine the influence of stem design on strain response at each individual key zone of interest. The *p* value obtained for each section corresponded to the likelihood that the difference in the mean principal strain for the implanted femur compared to that of the intact femur was due to chance. A *p*-value < 0.05 was deemed to give rejection with the preset statistical significance. Prior to the FE model validation, the corresponding fields of the DIC vs FE under the same loading were registered together in the ARAMIS environment. Nevertheless, following registration, the comparison points did not have identical locations. For this reason, polynomial of 4th degree approximations of DIC strain data were performed to overcome the exact measurement point mismatch between the two methods. Using the polynomial approximations, reported to the range of points x of the non-implanted FE model, we were able to perform linear correlation analyses for each separate case (intact bone, implanted bones with Minima S and Trilock BPS stem) and field of view (lateral and medial). The linear regression coefficient (slope and intercept) and the coefficient of determination (*R*^2^) were calculated for each analysis with 95% confidence bounds.

## Supplementary information

**Additional file 1.** Superimposition of the implant template-matched radiographs of the intact femurs on those of the implanted femurs.

## Data Availability

The datasets used and analyzed during the current study are available from the corresponding author on reasonable request.

## Abbreviations

THA: Total hip arthroplasty
DIC: Digital image correlation
FEA: Finite element analysis
FE: Finite element
BPS: Bone preservation stem
3D: Three-dimensional
SPSS: Statistical Package for Social Sciences
