# Supplementary material for: Comparative analysis of the biomechanical behavior of two different design metaphyseal-fitting short stems using digital image correlation
Source: Biomed Eng Online. 2020 Aug 19;19:65. doi: 10.1186/s12938-020-00806-y (PMC7437017; doi:10.1186/s12938-020-00806-y)
Supplement: Supplementary file 1 — Additional file 1. Superimposition of the implant template-matched radiographs of the intact femurs on those of the implanted femurs. [file 12938_2020_806_MOESM1_ESM.docx]

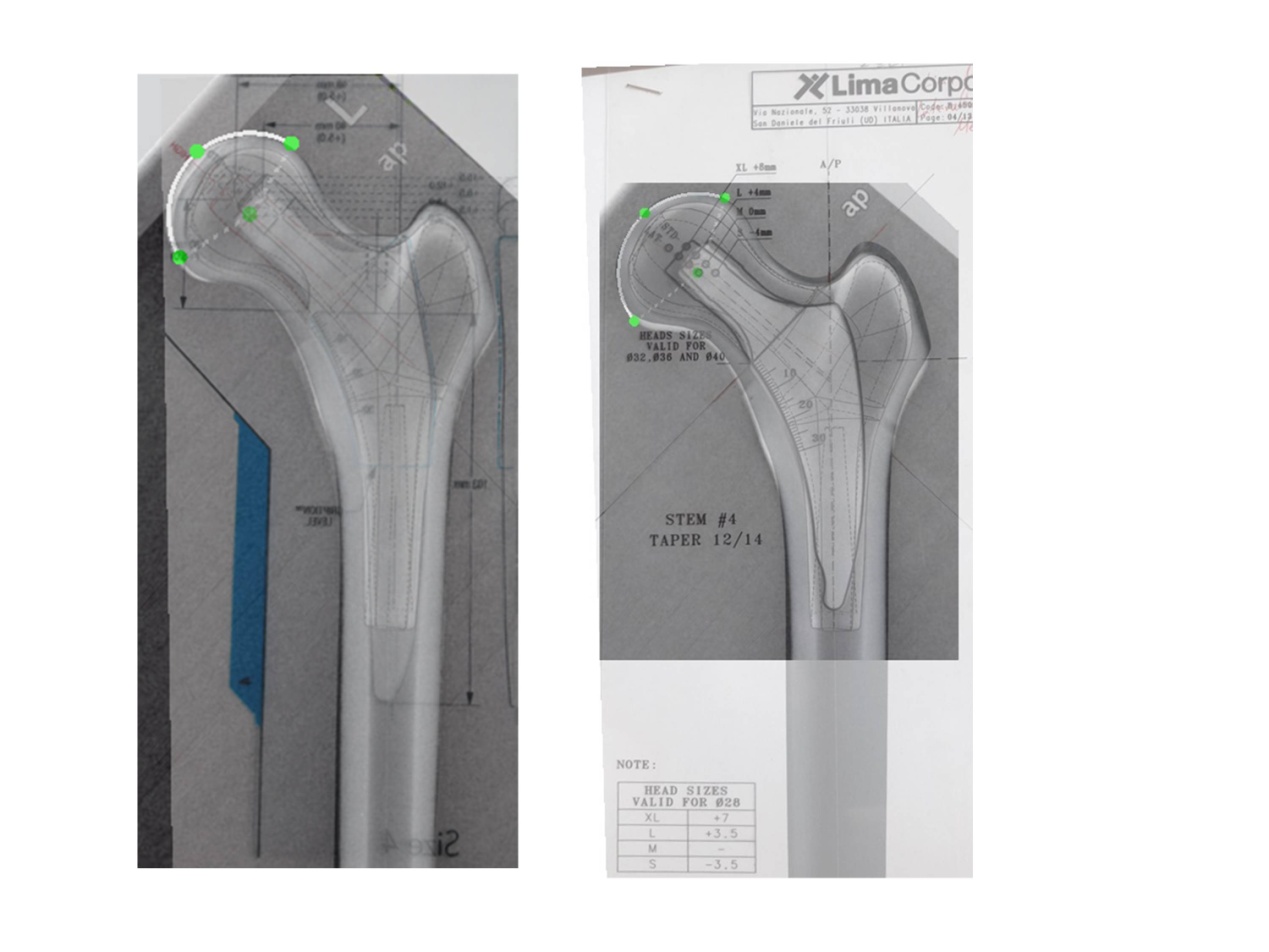


**B**

**A**

**Superimposition of the implant template-matched radiographs of the intact femurs on those of the implanted femurs**.

The accuracy of implantation of the femoral stems in the composite femurs in terms of correct implant size and positioning was verified using calibrated radiographs of the intact and implanted femurs, (A) Trilock BPS stem and (B) Minima S stem. The radiographs of the best fitting template-matched radiographs of the intact femurs were superimposed on those of the implanted femurs.
